# Supplementary material for: Measuring Dietary Intake of Pregnant Women Post-Bariatric Surgery: Do Women Meet Recommendations?
Source: Nutrients. 2025 Jan 14;17(2):285. doi: 10.3390/nu17020285 (PMC11767831; doi:10.3390/nu17020285)
Supplement: Supplementary file 1 [file nutrients-17-00285-s001.zip › nutrients-3384492-supplementary.pdf]

BACK MATTER

Supplementary materials

Table S1: Diet recalls completed at each gestational stage

24hour recalls completed	Enrolment (< week 23)	Week 28	Week 36
**1 recall**	5 (n=3)	7 (n=4)	17 (n=6)
**2 recalls**	8 (n=5)	13 (n=7)	8 (n=3)
**3 recalls**	89 (n=59)	80 (n=44)	75 (n=36)
**Total n=**	n=67	n=55	n=36

Figure S2: Relationship between dietetic care during pregnancy and reported nutrient intake. (a) Protein intake, (b) Carbohydrate intake, (c) Fat intake, (d) Saturated fat intake

Figure 4.1 Protein intake

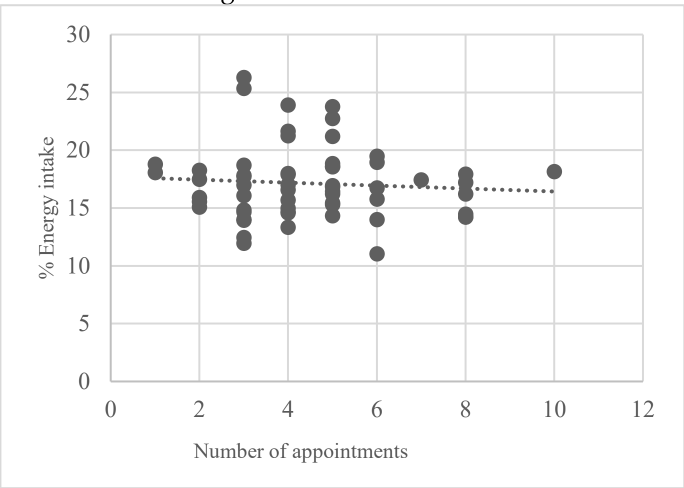

R=-0.078, p=0.556

Figure 4.2 Carbohydrate intake

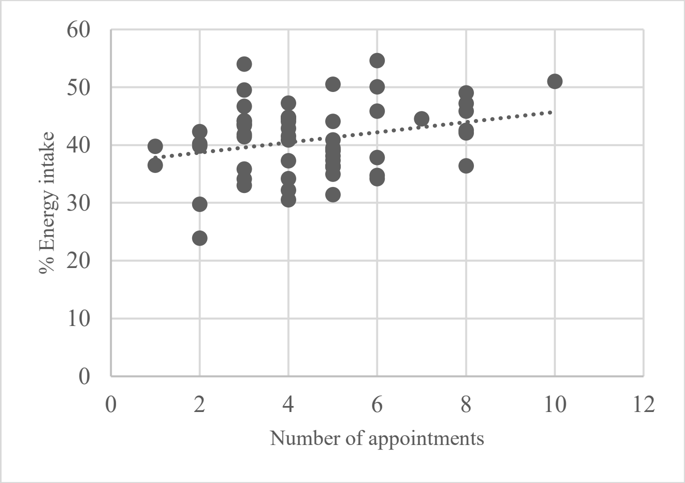

R=0.274, p=0.036

Figure 4.3 Fat intake

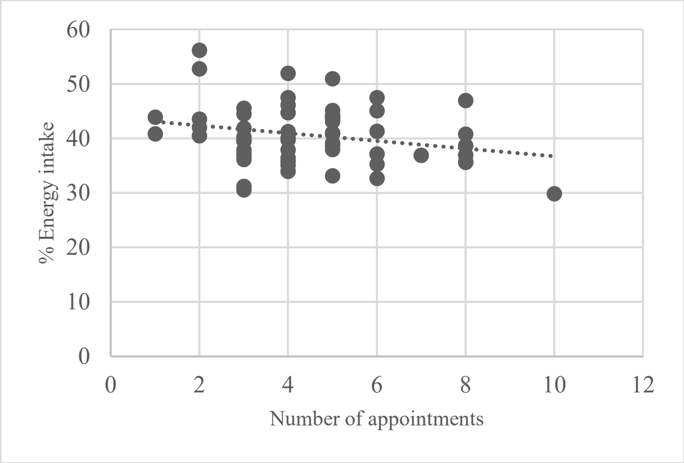

R=-0.251, p=0.055

Figure 4.4: Saturated fat intake

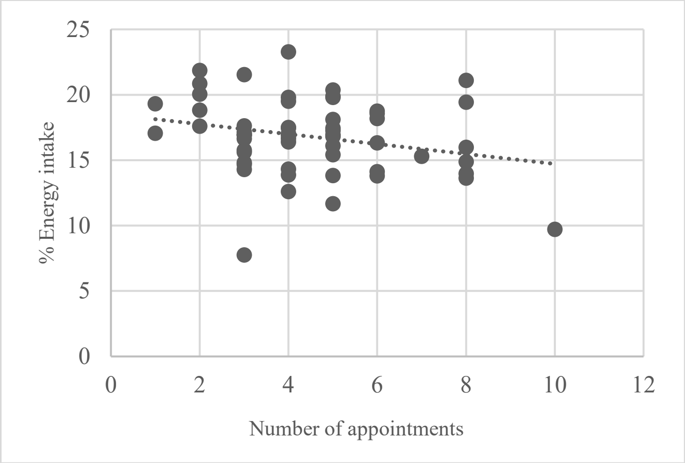

R=0.-0.255, p=0.051

Author Contributions

TG is the PhD student who led study design, data collection, analysis, and manuscript writing. HT, SdJ and SK provided supervision to all stages of the research, and contributed to manuscript preparation. SL and AK lead study recruitment at two sites and supported manuscript preparation. All authors read and approved the final manuscript.

Funding

This work has received no specific funding however Ms Guthrie receives PhD funding from the Royal Brisbane Women's Hospital. A/Prof de Jersey and A/Prof Alka Kothari are supported by a Metro North Health Clinician Research Fellowship.

Institutional Review Board Statement

The study was conducted according to the guidelines of the Declaration of Helsinki and approved by the Metro North Hospital and Health Services Human Research and Ethical Committee (EC00168) and the University of Queensland Human Ethics Committee on 19/10/2021.

Informed Consent Statement

Informed consent was obtained from all subjects involved in the study.

Acknowledgements

This study was made possible by the in-kind support of health professionals at several hospitals across Queensland, Australia. The authors would like to acknowledge and thank Windermere Lai, Candice Crellin, Helen Porteous and Dr Michelle Palmer (Logan Hospital), Kristen Riggs (Caboolture Hospital), Carolyn Berry and Christine McCourt (Redcliffe Hospital), Lisa McGuire, Deanne Rice and Amanda De La Cruz (Royal Brisbane Women's Hospital). The authors also wish to acknowledge the contribution

from William Pinzon Perez for statistical support received through the Research and Statistical Support Service, Faculty of Medicine, provided by QCIF Facility for Advanced Bioinformatics (QFAB), University of Queensland.

Conflict of interest

The authors declare no conflicts of interest in relation to this work.

References

1. Obesity Evidence Hub. *The disease burden of overweight, obesity and poor diet* 2021 [cited 2022 13/07/2022]; Available from: https://www.obesityevidencehub.org.au/collections/impacts/disease-burden-overweight-obesity-poor-diet.
2. National Health Service. *Health survey for England, 2019: Data tables* 2020; Available from: https://digital.nhs.uk/data-and-information/publications/statistical/health-survey-for-england/2019/health-survey-for-england-2019-data-tables.
3. Centre for Disease Control. *Women's Health*. 2019; Available from: https://www.cdc.gov/nchs/data/hus/2019/026-508.pdf.
4. Dağ, Z. and B. Dilbaz, Impact of obesity on infertility in women. *J Turk Ger Gynecol Assoc,* **2015**. *16*(2): p. 111-7.
5. Santos, S., et al., Impact of maternal body mass index and gestational weight gain on pregnancy complications: an individual participant data meta-analysis of European, North American and Australian cohorts. *BJOG,* **2019**. *126*(8): p. 984-995.
6. Langley-Evans, S.C., Nutrition in early life and the programming of adult disease: a review. **2015**. *28*(s1): p. 1-14.
7. Sim, K.A., S.R. Partridge, and A. Sainsbury, Does weight loss in overweight or obese women improve fertility treatment outcomes? A systematic review. *Obesity Reviews,* **2014**. *15*(10): p. 839-850.
8. Price, S.A.L., et al., Impact of preconception weight loss on fasting glucose and pregnancy outcomes in women with obesity: A randomized trial. *Obesity,* **2021**. *29*(9): p. 1445-1457.
9. Colquitt, J.L., et al., Surgery for weight loss in adults. *Cochrane Database of Systematic Reviews,* **2014**(8).
10. Australian Institute of Health and Welfare, *Weight loss surgery in Australia 2014-15: Australian hospital statistics*. 2017: Canberra.
11. Gulliford, M.C., et al., *Costs and outcoms of increasing access to bariatric surgery for obesity: cohort study and cost-effectiveness analysis using electronic health records*, in *Epidemiology of bariatric surgery in the UK*. 2016, MNIHR Journals Library: Southampton (UK)
12. American Society for Metabolic and Bariatric Surgery. *Estimate of Bariatric surgery numbers, 2011-2019* 2021 [cited 2021 20/10/2021]; Available from: https://asmbs.org/resources/estimate-of-bariatric-surgery-numbers.
13. Edison, E., et al., Bariatric Surgery in Obese Women of Reproductive Age Improves Conditions That Underlie Fertility and Pregnancy Outcomes: Retrospective Cohort Study of UK National Bariatric Surgery Registry (NBSR). *Obesity Surgery,* **2016**. *26*(12): p. 2837-2842.
14. Bariatric Surgery Registry. *Registry Data as at 31 December 2019* 2019 [cited 2021 20/10/2021]; Available from: https://www.monash.edu/__data/assets/pdf_file/0010/2158939/Bariatric-Surgery-Registry-Semi_Annual-Report_2019.pdf.
15. Mechanic, J.I., et al., Clinical practice guidelines for the perioperative nutrition, metabolic and nonsurgical support of patients undergoing bariatric procedures- 2019 update. *Endocrine Practice,* **2019**. *25*(12): p. 1346-1359.

16. Shawe, J., et al., Pregnancy after bariatric surgery: Consensus recommendations for periconception, antenatal and postnatal care. *Bariatric Surgery / Pregnancy,* **2019**. *20*(11): p. 1507-1522.
17. Ionut, V. and R.N. Bergman, Mechanisms responsible for excess weight loss after bariatric surgery. *J Diabetes Sci Technol,* **2011**. *5*(5): p. 1263-82.
18. Zarshenas, N., et al., The Relationship Between Bariatric Surgery and Diet Quality: a Systematic Review. *Obesity Surgery,* **2020**. *30*(5): p. 1768-1792.
19. Alkerwi, A., Diet quality concept. *Nutrition,* **2014**. *30*(6): p. 613-8.
20. Elder, K.A. and B.M. Wolfe, Bariatric Surgery: A Review of Procedures and Outcomes. *Gastroenterology,* **2007**. *132*(6): p. 2253-2271.
21. Banerjee, A., et al., The role of dumping syndrome in weight loss after gastric bypass surgery. *Surgical Endoscopy,* **2013**. *27*(5): p. 1573-1578.
22. Papamargaritis, D., et al., Dumping Symptoms and Incidence of Hypoglycaemia After Provocation Test at 6 and 12 Months After Laparoscopic Sleeve Gastrectomy. *Obesity Surgery,* **2012**. *22*(10): p. 1600-1606.
23. Chakhtoura, M.T., et al., Hypovitaminosis D in bariatric surgery: A systematic review of observational studies. *Metabolism,* **2016**. *65*(4): p. 574-585.
24. Lewis, C.A., et al., Does bariatric surgery cause vitamin A, B1, C or E deficiency? A systematic review. *Obesity Surgery,* **2018**. *28*(3): p. 3640-57.
25. O'Kane, M., et al., British Obesity and Metabolic Surgery Society Guidelines on perioperative and postoperative biochemical monitoring and micronutrient replacement for patients undergoing bariatric surgery—2020 update. *Obesity Reviews,* **2020**. *21*(11): p. e13087.
26. Mousa, A., A. Naqash, and S. Lim, Macronutrient and Micronutrient Intake during Pregnancy: An Overview of Recent Evidence. *Nutrients,* **2019**. *11*(2): p. 443.
27. Institute of Medicine., *Weight gain during pregnancy: reexamining the guidelines*. 2009, National Academic Press: Washington, DC.
28. Thompson, A.M. and J.A. Thompson, An evaluation of whether a gestational weight gain of 5 to 9 kg for obese women optimizes maternal and neonatal health risks. **2019**. *19*: p. 126.
29. Akhter, Z., et al., Pregnancy after bariatric surgery and adverse perinatal outcomes: A systematic review and meta-analysis. *PLoS Med,* **2019**. *16*(8): p. e1002866.
30. Al-Nimr, R.I., et al., Effects of Bariatric Surgery on Maternal and Infant Outcomes of Pregnancy—An Evidence Analysis Center Systematic Review. *Journal of the Academy of Nutrition and Dietetics,* **2019**. *119*(11): p. 1921-1943.
31. Galazis, N., et al., Maternal and neonatal outcomes in women undergoing bariatric surgery: a systematic review and meta-analysis. *European Journal of Obstetrics & Gynecology and Reproductive Biology,* **2014**. *181*: p. 45-53.
32. Kjaer, M.M. and L. Nilas, Pregnancy after bariatric surgery - a review of benefits and risks. *Acta Obstetricia et Gynecologica Scandinavica,* **2013**. *92*(3): p. 264-271.
33. Kwong, W., G. Tomlinson, and D.S. Feig, Maternal and neonatal outcomes after bariatric surgery; a systematic review and meta-analysis: do the benefits outweigh the risks? *American Journal of Obstetrics and Gynecology,* **2018**. *218*(6): p. 573-580.
34. Maggard, M.A., et al., Pregnancy and Fertility Following Bariatric Surgery. *JAMA,* **2008**. *300*(19): p. 2286.
35. Price, S.A., et al., Preconception management of women with obesity: A systematic review. *Obesity Reviews,* **2018**: p. 510-526.

36. Yi, X., et al., A meta-analysis of maternal and fetal outcomes of pregnancy after bariatric surgery. *International Journal of Obestetrics and Gynaecology,* **2015**. *130*(1): p. 3-9.
37. Guthrie, T.M., et al., A Systematic Review Investigating Maternal Nutrition During Pregnancy After Bariatric Surgery. *Obesity Surgery,* **2023**. *33*(6): p. 1857-1865.
38. Coupaye, M., et al., Impact of roux-en-Y gastric bypass and sleeve gastrectomy on fetal growth and relationship with maternal nutrition status. *Surgery for Obesity and Related Diseases,* **2018**. *14*: p. 1488-1494.
39. Queensland Clinical Guidelines, *Obesity and pregnancy (including post bariatric surgery)*. 2021, Department of Health.
40. Harris, P.A., et al., The REDCap consortium: Building an international community of software platform partners. *Journal of Biomedical Informatics,* **2019**. *95*: p. 103208.
41. Harris, P.A., et al., Research electronic data capture (REDCap)--a metadata-driven methodology and workflow process for providing translational research informatics support. *J Biomed Inform,* **2009**. *42*(2): p. 377-81.
42. Svarstad, B.L., et al., The brief medication questionnaire: A tool for screening patient adherence and barriers to adherence. *Patient Education and Counseling,* **1999**. *37*(2): p. 113-124.
43. Sanda, B., et al., Reliability and concurrent validity of the International Physical Activity Questionnaire short form among pregnant women. *BMC Sports Science, Medicine and Rehabilitation,* **2017**. *9*(7).
44. Moshfegh, A.J., et al., The US Department of Agriculture Automated Multiple-Pass Method reduces bias in the collection of energy intakes. *The American Journal of Clinical Nutrition,* **2008**. *88*(2): p. 324-332.
45. National Academies of Sciences, E., and Medicine ,Health and Medicine Division; Food and Nutrition Board; Committee on the Dietary Reference Intakes for Energy, , *Dietary Reference Intakes for Energy*. 2005, Washington (DC): National Academies Press (US).
46. Chen, H.L., *A simple easy-to-use spreadsheet for automatic scoring of the International Physical Activity Questionnaire (IPAQ) Short Form (updated November 2016)*. 2016, ResearchGate.
47. Council, N.H.a.M.R., *Nutrient Reference Values for Australia and New Zealand Including Recommended Dietary Intakes*, N.Z.M.o.H. Australian Government Department of Health and Ageing, Editor. 2006, National Health and Medical Research Council: Canberra.
48. Subar, A.F., et al., The Automated Self-Administered 24-Hour Dietary Recall (ASA24): A Resource for Researchers, Clinicians, and Educators from the National Cancer Institute. *Journal of the Academy of Nutrition and Dietetics,* **2012**. *112*(8): p. 1134-1137.
49. Blumfield, M.L., et al., Systematic review and meta-analysis of energy and macronutrient intakes during pregnancy in developed countries. *Nutr Rev,* **2012**. *70*(6): p. 322-336.
50. Butte, N.F. and J.C. King, Energy requirements during pregnancy and lactation. *Public Health Nutr,* **2005**. *8*(7a): p. 1010-1027.
51. Most, J., et al., Energy Intake Requirements in Pregnancy. *Nutrients,* **2019**. *11*(8): p. 1812.
52. Khaire, A., et al., Maternal fats and pregnancy complications: Implications for long-term health. *Prostaglandins, Leukotrienes and Essential Fatty Acids,* **2020**. *157*: p. 102098.
53. Crume, T.L., et al., Maternal dietary intake during pregnancy and offspring body composition: The Healthy Start Study. *American Journal of Obstetrics and Gynecology,* **2016**. *215*(5): p. 609.e1-609.e8.

54. Maslova, E., et al., Maternal intake of fat in pregnancy and offspring metabolic health – A prospective study with 20 years of follow-up. *Clin Nutr,* **2016**. *35*(2): p. 475-483.
55. Hilaire, M.J., et al., The association of higher offspring early-childhood weight gain with prepregnancy metabolic and bariatric surgery. *Obesity (Silver Spring),* **2024**. *32*(11): p. 2012-2023.
56. Moradi, R., et al., The Effect of Maternal Bariatric Surgery on Offspring Anthropometry: A Mixed Cohort. *Obesity Surgery,* **2024**. *34*(9): p. 3229-3235.
57. Reijonen, J.K., et al., Association of dietary fiber, liquid intake and lifestyle characteristics with gastrointestinal symptoms and pregnancy outcome. *European journal of obstetrics & gynecology and reproductive biology. X,* **2022**. *16*: p. 100168-100168.
58. De-Regil, L.M., et al., Effects and safety of periconceptional oral folate supplementation for preventing birth defects. *Cochrane Database of Systematic Reviews,* **2015**(12).
59. McGuire, E., Nutritional consequences of bariatric surgery for pregnancy and breastfeeding. *Breastfeeding Review,* **2018**. *26*(3): p. 19-26.
60. Palacios, C., L.K. Kostiuk, and J.P. Peña-Rosas, Vitamin D supplementation for women during pregnancy. *Cochrane Database of Systematic Reviews,* **2019**(7).
61. Chan, L., Q. Zheng, and T. Colovos, Assessing micronutrient adherence after bariatric surgery: an exploratory study. *Journal of Obesity and Bariatrics,* **2015**. *2*(2): p. 1-8.
62. Elder-Robinson, E.C., et al., Survey of Australian clinicians' antenatal care and management practices in pregnant women with a history of bariatric surgery. *Obstetric Medicine,* **2023**. *16*(2): p. 88-97.
63. Sweet, L. and V. Vasilevski, Women's experiences of pregnancy and lactation after bariatric surgery: A scoping review. *Midwifery,* **2022**. *110*: p. 103338.
64. Vasilevski, V., et al., Experiences and information needs of women who become pregnant after bariatric surgery: An interpretive descriptive qualitative study. *Midwifery,* **2023**. *121*: p. 103652.
65. Smid, M.C., et al., Pregnancy After Bariatric Surgery: National Survey of Obstetrician's Comfort, Knowledge, and Practice Patterns. *Obes Surg,* **2017**. *27*(9): p. 2354-2359.
66. Black, A.E. and T.J. Cole, Biased Over- Or Under-Reporting is Characteristic of Individuals Whether Over Time or by Different Assessment Methods. *Journal of the American Dietetic Association,* **2001**. *101*(1): p. 70-80.
67. Poslusna, K., et al., Misreporting of energy and micronutrient intake estimated by food records and 24 hour recalls, control and adjustment methods in practice. *British Journal of Nutrition,* **2009**. *101*(S2): p. S73-S85.
